# Gap junctions in the ovary of *Drosophila melanogaster*: localization of innexins 1, 2, 3 and 4 and evidence for intercellular communication via innexin-2 containing channels

**DOI:** 10.1186/1471-213X-8-111

**Published:** 2008-11-27

**Authors:** Johannes Bohrmann, Jennifer Zimmermann

**Affiliations:** 1 RWTH Aachen University, Institut für Biologie II, Abt. Zoologie und Humanbiologie, Kopernikusstrasse 16, 52056 Aachen, Germany

## Abstract

**Background:**

In the *Drosophila *ovary, germ-line and soma cells are interconnected via gap junctions. The main gap-junction proteins in invertebrates are members of the innexin family. In order to reveal the role that innexins play in cell-cell communication during oogenesis, we investigated the localization of innexins 1, 2, 3 and 4 using immunohistochemistry, and analyzed follicle development following channel blockade.

**Results:**

We found innexin 1 predominantly localized to the baso-lateral domain of follicle cells, whereas innexin 2 is positioned apico-laterally as well as apically between follicle cells and germ-line cells. Innexin 3 was observed laterally in follicle cells and also in nurse cells, and innexin 4 was detected in the oolemma up to stage 8 and in nurse-cell membranes up to stage 12. In order to test whether innexins form channels suitable for intercellular communication, we microinjected innexin antibodies in combination with a fluorescent tracer into the oocyte of stage-10 follicles. We found that dye-coupling between oocyte and follicle cells was largely reduced by innexin-2 antibodies directed against the intracellular C-terminus as well as against the intracellular loop. Analyzing in vitro, between stages 10 and 14, the developmental capacities of follicles following microinjections of innexin-2 antibodies revealed defects in follicle-cell differentiation, nurse-cell regression, oocyte growth and choriogenesis.

**Conclusion:**

Our results suggest that all analyzed innexins are involved in the formation of gap junctions in the ovary. While innexins 2 and 3 are colocalized between soma cells, innexins 2 and 4 are colocalized between soma and germ-line cells. Innexin 2 is participating in cell-cell communication via hemichannels residing in the oolemma. It is obvious that gap-junctional communication between germ-line and soma cells is essential for several processes during oogenesis.

## Background

During *Drosophila *oogenesis, germ-line and soma cells are known to interact intimately with each other, for example concerning follicle organization, vitellogenesis and egg-shell production [1-3]. In many cases, however, the exact routes of information exchange have remained elusive. During establishment of the dorsoventral and anteroposterior axes, intercellular communication between germ-line and soma cells has been shown to be essential as well. Besides other mechanisms, e.g. contact of membrane receptors and their ligands [4-6], the transfer of small molecules via gap junctions might be involved in such communication processes.

In recent years, gap-junction mediated cell-cell communication in *Drosophila *has gained growing attention, as several mutants have been described in which specific developmental defects are correlated with altered gap-junction plaques or channels [7-14]. In arthropods, the coordination of physiological and developmental processes by means of gap-junctional communication [15-19] might be even more important than in vertebrates, since the channels of arthropods allow the exchange of molecules of up to 3000 D [20]. In special cases, even much larger molecules, e.g. calmodulin, seem to be able to pass through gap junctions [21,22]. Recently, it has been reported that gene expression can be regulated by neighbouring cells through the exchange of siRNAs via gap junctions [23].

While vertebrates use connexins for the assembly of gap-junction channels (connexons) [24,25], the main gap-junction proteins found in invertebrates are members of the innexin family [26-29]. However, additional proteins have been detected: the innexin-related pannexins [30] and the ductins [31-33]. Neither innexins nor pannexins have homologies with either connexins or ductins. While pannexins were found only in vertebrates, ductins were observed in gap junctions of vertebrates as well as invertebrates. In some studies on insect gap junctions, further proteins have been deteced [26].

**Figure 1:**
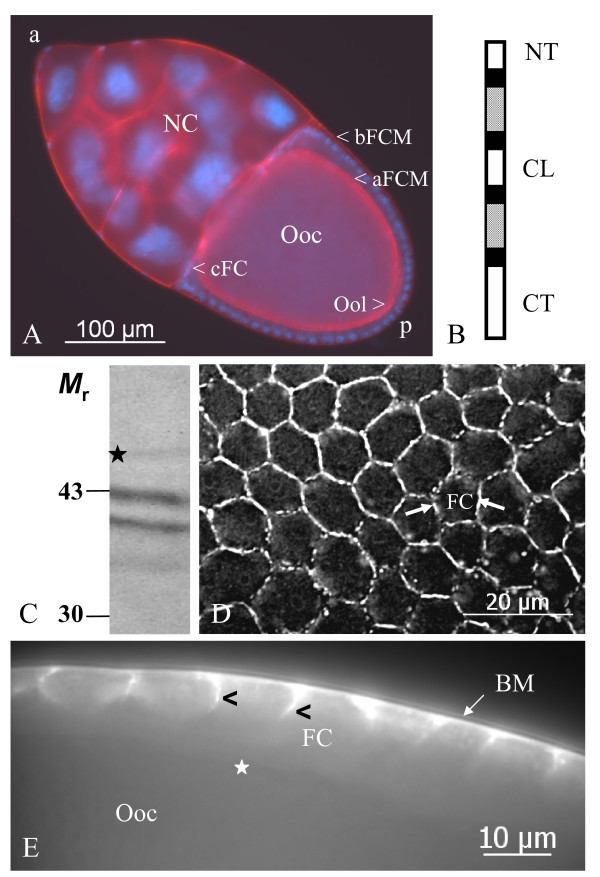
**Inx1 is localized to baso-lateral follicle-cell membranes**. A: *Drosophila *stage-10b follicle, showing different cell types and membrane regions relevant for the present study (blue: nuclei, stained with DAPI; red: F-actin, stained with rhodaminylphalloidin). B: Scheme of the predicted protein structure of innexins (white: intracellular domains (NT, N-terminus; CL, cytopasmic loop; CT, C-terminus), black: transmembrane domains, grey: extracellular domains). Hemichannels made of six molecules can be either homo- or heteromeric, channels made of two hemichannels either homo- or heterotypic. C: On immunoblots of ovary extracts using Inx1-antiserum (AInx1Rb-CT) a weak band at the calculated molecular mass of 46 kDa (asterisk) and two bands at 43 and 41 kDa are recognized. D: Inx1 is detected in a continuous pattern as well as in presumed gap-junction plaques at the lateral follicle-cell membranes (FC, arrows, stage 10a, surface view, WFF). E: Inx1 is restricted to the baso-lateral domain (arrowheads) of the follicle cells, i. e. the apical domain and the oolemma (asterisk) is not stained (stage 10a, optical median section, WFF). a, anterior; aFCM, apical follicle-cell membrane; bFCM, basal follicle-cell membrane; BM, basement membrane; cFC, centripetally migrating follicle cells; NC, nurse cells; Ooc, oocyte; Ool, oolemma.

The ovarian follicle of *Drosophila *consists of a group of 16 germ-line cells surrounded by a layer of somatic follicle cells (Fig. 1A). The oocyte and its 15 nurse cells form a cytoplasmic continuum via intercellular bridges as well as via gap junctions, and the same holds true for the follicle cells. With the germ-line cells, however, the follicle cells are only connected via gap junctions, that have been found to vary in structure and size during the course of oogenesis [34,35]. By way of the intercellular distribution of microinjected fluorescent tracers, we have previously revealed stage-specific communication between oocyte and follicle cells. A variety of treatments has been found to either inhibit or to stimulate dye-coupling, e.g. acidic pH, high intracellular [Ca^2+^], octanol, dinitrophenol, a juvenile hormone analogue or 20-hydroxyecdysone [36].

Of the eight known *Drosophila *innexins, the mRNAs of innexins 1, 2, 3, 4 and 7 were detected in the ovary [37]. So far, only for innexin 4 the localization and functioning of the protein during early oogenesis have been analyzed [13,38]. We investigated by immunoblots and immunohistochemistry the occurrence and detailed localization of innexins 1, 2, 3 and 4 throughout oogenesis. Our results suggest that all analyzed innexins are involved in the formation of ovarian gap junctions.

In order to test whether innexins form intercellular communication channels in the ovary, we microinjected various innexin antisera in combination with the fluorescent tracer Lucifer Yellow into the oocyte of stage-10 follicles. We found that only innexin-2 antibodies were able to significantly reduce the dye-coupling observed between oocyte and follicle cells. Therefore, innexin 2 is involved in cell-cell communication between germ-line and soma cells by participating in gap-junction hemichannels residing in the oolemma.

We further analyzed the developmental capacities of follicles following microinjections of innexin-2 antibodies. Since oogenesis in vitro was affected in a significant fraction of microinjected follicles, we conclude that cell-cell communication via innexin-2-containing gap junctions is essential for various processes of normal follicle development through stages 10 to 14.

## Methods

### Antisera

For immunocytochemical and immunoblot analysis as well as for microinjections, we used the following anti-peptide sera, which have been raised against cytoplasmic regions (c.f. Fig. 1B) of different innexins from *Drosophila melanogaster*: (1) a rabbit antiserum to the C-terminus of innexin 1 (AInx1Rb-CT) [14,39], (2) two rabbit antisera to different regions of the C-terminus of innexin 2 (AInx2Rb-CT(KLRH) and AInx2Rb-CT(REM) [14,39]), (3) a guinea-pig antiserum to the cytoplasmic loop of innexin 2 (AInx2GP-CL, R. Bauer, pers. com.), (4) a guinea-pig antiserum to the C-terminus of innexin 3 (AInx3GP-CT, R. Bauer, pers. com.), (5) a rabbit antiserum to the cytoplasmic loop of innexin 3 (AInx3Rb-CL) [40], (6) a rabbit antiserum to the C-terminus of innexin 4 (AInx4Rb-CT) [13,38], and (7) as a control, a rabbit non-immune serum (NIS). The antisera to innexins 1, 2 and 3 were kindly provided by R. Bauer and M. Hoch (Bonn, Germany), whilst the antiserum to innexin 4 was a gift from S. Tazuke (Philadelphia, PA, USA).

### Preparation of follicles

*Drosophila melanogaster *wild-type Oregon R flies were reared at about 20°C on standard food with additional fresh yeast. Individual females of 2–3 days old were killed by crushing the thorax with tweezers without previous etherization or chilling. For detailed analysis, follicles of various stages [41] were dissected out of ovaries in *Drosophila *PBS (DPBS) [42,43] using tungsten needles.

### Immunoblotting

Immunoblots were performed as described previously [31]. In short, homogenates of ovaries were sonicated and briefly boiled. The proteins were separated using 12% SDS-PAGE and transferred to nitrocellulose membranes. Nonspecific binding sites were blocked with 5% skimmed milk powder/PBS and the blots were incubated (1) in 1% BSA/PBS (control), (2) in AInx1Rb-CT (diluted 1:400 with 1% BSA/PBS), (3) in AInx2Rb-CT(KLRH, diluted 1:400), (4) in AInx2GP-CL (diluted 1:200), (5) in AInx3GP-CT (diluted 1:200), (6) in AInx3Rb-CL (diluted 1:400), and (7) in AInx4Rb-CT (diluted 1:1000), respectively. Subsequently, the appropriate biotinylated secondary antibodies (goat-anti-rabbit IgG or goat-anti-guinea-pig IgG, Jackson, PA, USA; diluted 1:1000), streptavidin-peroxidase (Dianova, Germany; diluted 1:1000) and H_2_O_2_/4-chloro-1-naphthol (Sigma, Germany) were applied, and photographs were taken using a digital camera. Each experiment was performed at least three times.

### Indirect immunofluorescence preparations

For immunostaining, follicles were fixed for 30 minutes at 4°C in 4% formaldehyde dissolved in PBS, washed in PBS and blocked for 1 hour at 20°C with 2% BSA/0.1% Triton X-100/PBS. Thereafter, the follicles were incubated overnight at 4°C (1) in 0.5% BSA/0.1% Triton X-100/PBS (control), (2) in NIS diluted 1:200 with 0.5% BSA/0.1% Triton X-100/PBS (control), (3) in AInx1Rb-CT (diluted 1:50), (4) in AInx2Rb-CT(REM, diluted 1:50), (5) in AInx2GP-CL (diluted 1:20), (6) in AInx3GP-CT (diluted 1:20), (7) in AInx3Rb-CL (diluted 1:50), and (8) in AInx4Rb-CT (diluted 1:4000), respectively.

After washing 6 times for 10 min, the follicles were either treated with a 1:100 dilution of biotinylated goat-anti-rabbit IgG or with a 1:100 dilution of biotinylated goat-anti-guinea-pig IgG (Jackson) for 1 h at 20°C, washed again 6 times and incubated in a 1:1000 dilution of streptavidin-Texas Red (Dianova) for 30 min. For double-staining experiments, a 1:2000 dilution of goat-anti-rabbit-Cy3 (Jackson) as well as a 1:100 dilution of donkey-anti-guinea-pig-FP488 (FluoProbes, Interchim, France) were used for 1 h at 20°C. Washing was repeated 6 times, and the nuclei were stained with 0.2 μg/ml DAPI (Sigma) in PBS for 3 min. Thereafter, the follicles were either mounted in Fluoromount G (Interchim) and viewed in a Zeiss Axiovert 200 wide-field fluorescence (WFF) microscope equipped with a Hamamatsu Orca ER camera, or they were mounted in glycerine/PBS 1:1 and viewed in a Leica DMRE laser-scanning microscope (LSM). Each experiment was performed at least three times.

### Microinjection procedure and analysis of dye-coupling

Stage-10 follicles, in which the oocyte occupies about 1/2 of the follicle's volume, were carefully isolated in R-14 medium [42,43]. Samples of 5–10 follicles lacking any signs of injury were washed in R-14 and immediately transferred to the microinjection chamber. The microinjection procedure was described previously [36,44]. In short, micropipettes were pulled from 1-mm glass capillaries containing a filament, and microinjections were carried out on an inverted epifluorescence microscope (Zeiss Axiovert 200). The injection pipette (tip diameter 1–2 μm) was mounted on a motorized micromanipulator equipped with a piezo translator (Märzhäuser, PM 20), and coupled to a microinjector (NPI, PDES-O2T). The recipient follicle was held in place with a suction pipette (tip diameter 30–50 μm) mounted on a second micromanipulator, and coupled to a screw-adjustable syringe. The volume injected into each oocyte (using a pressure of about 300 hPa) was in the order of 100 pl, which is equivalent to about 5% of the oocyte volume [45].

In control experiments, the fluorescent tracer Lucifer Yellow CH (LY, Sigma; 2.5% solution (w/v) in distilled water) and NIS (diluted 1:5 with distilled water and mixed 1:1 with 5% LY-solution), respectively, were used. In further experiments the following antisera (mixed 1:1 with 5% LY-solution) were microinjected: (1) AInx1Rb-CT, (2) AInx2Rb-CT(KLRH), (3) AInx2Rb-CT(REM)), (4) AInx2GP-CL, (5) AInx3GP-CT, (6) AInx3Rb-CL, and (7) AInx4Rb-CT, respectively. Using the Hamamatsu Orca ER camera, very weak levels of fluorescence were detectable in the follicular epithelium. In Table 1, the results of six experiments were pooled. Statistical significance of differences between relative frequencies of dye-couplig was established at the α = 0.05 level using the *Χ*^2^-test.

**Table 1:** Summary of experiments analyzing dye-coupling between oocyte and follicle cells

Microinjected solution	*n *injected for dye-coupling	% follicles with dye-coupling
H_2_O dest.	13	85
NIS	40	48
AInx1Rb-CT	34	50
AInx2Rb-CT(KLRH)	39	17*
AInx2Rb-CT(REM)	39	38
AInx2GP-CL	53	30*
AInx3GP-CT	38	37
AInx3Rb-CL	20	40
AInx4Rb-CT	22	46

*, significantly different from NIS (*P *< 0.05); *n*, number of injected stage-10 follicles; for further details, see text

### In-vitro development of microinjected follicles

Microinjections were carried out as described above. In-vitro culture of follicles through stages 10b to 14 was performed in small alcohol-cleansed glass blocks containing 100 μl R-14 medium covered by an air volume of about 1 ml and a glass slide at 20°C in a wet chamber [43,45]. In order to test for the effects of gap-junctional blockade on follicle development, the antisera AInx2Rb-CT(KLRH) and AInx2GP-CL were microinjected into the oocyte. Non-injected follicles, follicles injected with distilled water, follicles injected with NIS and follicles injected with AInx4Rb-CT served as controls. Photographs were taken following microinjections at stage 10b and after 8 hours using a Nikon SMZ1000 stereomicroscope equipped with a Canon digital camera. Staging of follicles developed in vitro was performed as described before [45]. In Table 2, the results of four experiments were pooled. For statistical evaluation the *Χ*^2^-test (α = 0.05) was used.

**Table 2:** Summary of experiments analyzing in-vitro development

Microinjected solution	*n *injected for in-vitro development	% follicles developed to stages 12–14
n. i.	8	88
H_2_O dest.	45	70
NIS	20	55
AInx2Rb-CT(KLRH)	50	24*
AInx2GP-CL	11	27*
AInx4Rb-CT	14	50

*, significantly different from NIS (*P *< 0.05); *n*, number of injected stage-10b follicles; for further details, see text

## Results

### Detection of innexins in the ovary by immunoblotting

Using immunoblots, we analyzed whether the mRNAs of innexins 1, 2, 3 and 4, which have been described in the *Drosophila *ovary [37], become translated during oogenesis. In ovarian extracts, some of the used innexin antisera, that have all been characterized previously ([13,14,38-40] and R. Bauer, pers. com.), revealed only weak bands at the molecular masses calculated from the amino-acid sequences of the proteins. But further bands at lower molecular masses were always detected, which are specific for the respective innexin (Inx), and which have been observed in embryonic extracts too (not shown).

**Figure 2:**
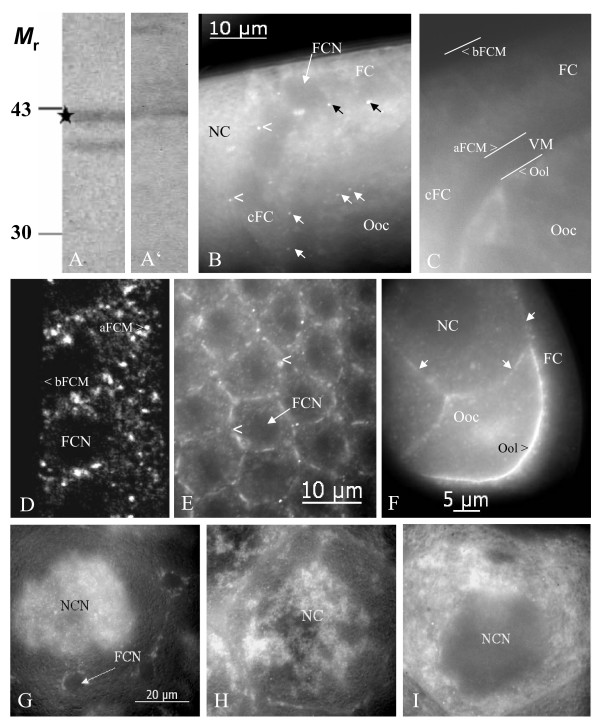
**Inx2 is localized to membrane plaques in follicle cells and germ-line cells and is distributed stage-specifically in nurse cells**. On immunoblots of ovary extracts using Inx2-antisera (A: AInx2Rb-CT(KLRH), A': AInx2GP-CL) a band at the calculated molecular mass of 42 kDa (asterisk) and a band at 39 kDa are recognized. B: Inx2 is localized to presumed gap-junction plaques between follicle cells as well as between follicle cells and germ-line cells (stage 10b, AInx2Rb-CT(REM), optical median section, WFF; see also Fig. 4D). Black arrows point to apico-laterally situated plaques between follicle cells, white arrows point to plaques between follicle cells and oocyte, and arrowheads point to plaques between centripetally migrating follicle cells and nurse cells. C: Control follicle (NIS) showing the anterior-dorsal region of the oocyte and the follicular epithelium (comparable to the region shown in B). Different membrane regions are marked with white lines. Apical follicle-cell membranes make contact with the oolemma via microvilli spanning the developing vitelline membrane (VM). D, E: Inx2 is found in lateral and apical, but not in basal follicle-cell membranes (arrowheads, stage 11, Anti-Inx2GP-CL, D: LSM, E: optical tangential section, WFF). F: Inx 2 is located in the oolemma (arrowhead) as well as in nurse-cell membranes (white arrows, stage 8, Anti-Inx2GP-CL, optical median section, WFF). G-I: Stage-specific distribution of Inx2 in the nurse cells (AInx2Rb-CT(REM), WFF): During stage 10a (G), Inx2 accumulates around nurse-cell nuclei (NCN). During stage 10b (H), Inx2 becomes dispersed in the cytoplasm. During nurse-cell regression (stage 11, I), Inx2 is found in cytoplasmic clouds and in particles that become transported into the oocyte. FCN, follicle-cell nucleus (not stained); for further abbreviations, see legend to Fig. 1.

**Figure 3:**
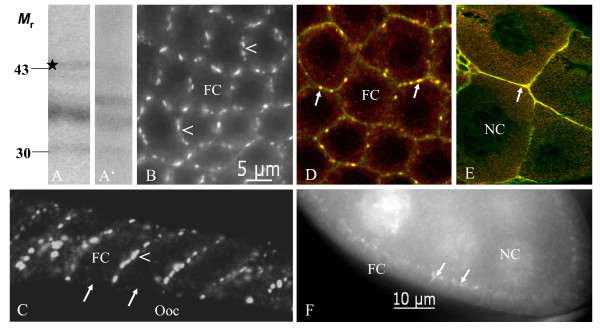
**Inx3 is colocalized with Inx2 to membrane plaques in follicle cells and nurse cells**. On immunoblots of ovary extracts using Inx3-antisera (A: AInx3GP-CT, A': AInx3Rb-CL) a weak band at the calculated molecular mass of 45 kDa (asterisk) and two bands at 35 and 30 kDa are recognized. B, C: Inx3 is localized to presumed gap-junction plaques between follicle cells (stage 10a). It is expressed in a continuous as well as punctate pattern at the lateral membranes (arrowheads, B: AInx3Rb-CL, WFF, C: AInx3GP-CT, LSM), but is missing at the apical membranes and at the oolemma (arrows). D, E: Inx2 (red, AInx2Rb-CT(REM)) and Inx3 (green, AInx3GP-CT) are colocalized in lateral follicle-cell membranes (yellow, arrows in D, stage 10a, LSM) as well as in nurse-cell membranes (yellow, arrow in E, stage 10b, LSM). F: In young follicles, Inx3 is located in plaques between nurse cells and follicle cells (arrows) and around nurse-cell nuclei (stage 7, AInx3Rb-CL, WFF). For further abbreviations, see legend to Fig. 1.

The antiserum AInx1Rb-CT against the C-terminus of Inx1 (for different protein domains, see Fig. 1B) recognized a weak band at the calculateded molecular mass of 46 kDa (Fig. 1C; also R. Bauer, pers. com.). Two Inx2-antisera, AInx2Rb-CT(KLRH) against the C-terminus and AInx2GP-CL against the cytoplasmic loop, both recognized bands at the calculateded molecular mass of 42 kDa (Fig. 2A, A'; also [14]), whereas the reaction of AInx2Rb-CT(REM) was very weak (not shown; also R. Bauer, pers. com.). Both Inx3-antisera, AInx3GP-CT against the C-terminus and AInx3Rb-CL against the cytoplasmic loop, recognized bands at the calculateded molecular mass of 45 kDa (Fig. 3A, A'; also [40]). The antiserum AInx4Rb-CT against the C-terminus of Inx4 recognized a very weak band at the calculateded molecular mass of 43 kDa (not shown; also [13,38]).

### Localization of innexins during oogenesis by immunocytochemistry

Using indirect immunofluorescence microscopy, we investigated the cellular localization of innexins 1, 2, 3 and 4 during the course of *Drosophila *oogenesis. For orientation, an overview of a stage-10b follicle is shown in Fig. 1A. All antisera clearly recognized cytoplasmic as well as membranous antigens, with the exception of AInx2Rb-CT(KLRH), which appeared to be less suitable for whole-mount immunocytochemistry (but was optimal for SL2 cells; R. Bauer, pers. com.). Punctate membrane labeling is presumed to represent gap-junction plaques, while diffuse or continuous membrane labeling represents either homogeniously dispersed channels or hemichannels. Labeling of cytoplasmic clouds or particles is presumed to represent channel precursors. The results presented below are specific, since in control preparations, incubated without primary antibodies, no staining was observed (e.g. Fig. 2C).

**Figure 4:**
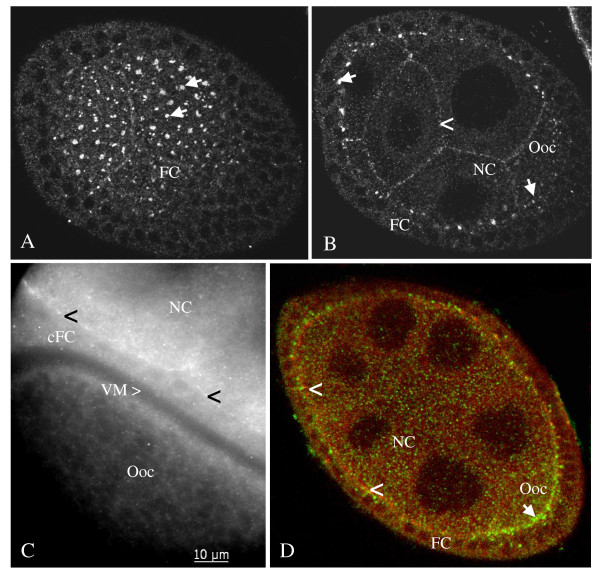
**Inx4 is localized to membrane plaques in germ-line cells and seems to interact with Inx2 in follicle cells**. A, B: Inx4-plaques (arrows in A (tangential section) and B (median section), stage 6, LSM) were detected between each follicle cell and the underlying oocyte and nurse cells, respectively. Continuous staining was found at the nurse-cell membranes (arrowhead in B). C: Inx4-plaques (black arrowheads, stage 10b, WFF) are located between nurse cells and the centripetally migrating follicle cells. D: Inx2 in follicle-cell membranes (green, AInx2GP-CL) and Inx4 in nurse-cell membranes (red, AInx4Rb-CT) are in close vicinity (arrowheads, stage 8, LSM) and seem to interact. Beginning with stage 8, only Inx2 is present between oocyte and follicle cells (arrow). For further abbreviations, see legend to Fig. 1.

We found Inx1 predominantly localized to the baso-lateral domain of follicle cells (Fig. 1D, E), whereas in germ-line cells no membrane labeling was detected. Inx2, on the other hand, is positioned apico-laterally between follicle cells as well as apically between follicle cells and oocyte and also between follicle cells and nurse cells (Fig. 2B–F). Inx2-labeling is present in the oolemma (Figs. 2F, 4D) and in the nurse-cell membranes (Figs. 2F, 3E). During stages 10 and 11, when the apical follicle-cell membranes are connected with the oolemma via gap junctions located on microvilli spanning the developing vitelline membrane [41], Inx2-plaques are found in this region (Fig. 2B–D). In the nurse cells, the distribution of Inx2 changed stage-specifically, starting with diffuse localization around the nuclei in stage 10a (Fig. 2G). During stages 10b and 11, Inx2 is observed in cytoplasmic clouds and particles (Fig. 2H, I), which become delivered into the growing oocyte during nurse-cell regression [41].

Inx3, which has been shown to heteromerize with Inx2 in embryonic epithelia [40], is colocalized with Inx2 in nurse-cell membranes as well as in lateral, but not in apical, membranes of follicle cells covering the oocyte (Fig. 3B–E). This contrasts with its lateral as well as apical appearance in follicle cells covering the nurse cells (Fig. 3F). In nurse cells, membrane labeling (stage 10, Fig. 3E) and cytoplasmic labeling around the nuclei (stage 7, Fig. 3F) was observed for Inx3.

Inx4 is known to be germ-line specific [13,37,38]. We detected Inx4-labelling in the oolemma up to stage 8 and in the nurse-cell membranes up to stage 12. While continuous labeling was observed between nurse cells, characteristic plaques were found up to stage 9 in the germ-line cell membranes beneath each single follicle cell (Fig. 4A, B; c.f. [13]). In these membrane plaques, Inx4 in germ-line cells appears to be in close vicinity to Inx2 in follicle cells (Fig. 4D). Inx4-plaques are also present between nurse cells and centripetally migrating follicle cells (stage 10b, Fig. 4C). Beginning with vitellogenesis (stage 8), only Inx2, but not Inx4, was detected in the oolemma (Fig. 4D).

### Analysis of dye-coupling following microinjections of innexin antisera

A critical test for the involvement of Inx2 in intercellular communication between oocyte and follicle cells is the specific blockade of dye-coupling with antibodies directed against cytoplasmic regions of Inx2. We microinjected various antisera in combination with the fluorescent tracer Lucifer Yellow CH into the oocyte. For technical reasons, the youngest follicles that can be successfully microinjected are of stage 8 [36]. We used stage-10 follicles in the present experiment. Since, beginning with stage 8, only Inx2 was detectable in the oolemma (see above), antisera directed against other innexins served as controls (Table 1).

**Figure 5:**
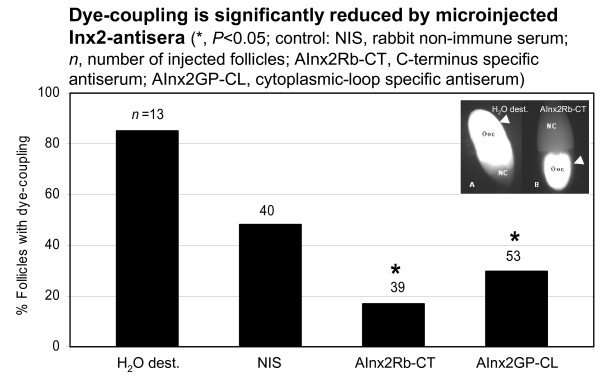
**Microinjected Inx2-antisera inhibit communication between oocyte and follicle cells**. In 85% (H_2_O dest.) and 48% (NIS), respectively, of the control follicles (stage 10), Lucifer Yellow CH was found to move from the microinjected oocyte (Ooc) into the nurse cells (NC) as well as into the surrounding follicle cells (arrowhead in inset A). In contrast, in 83% (AInx2Rb-CT) and 70% (AInx2GP-CL), respectively, of the follicles microinjected with Inx2-antisera, gap-junctional communication between oocyte and follicle cells was blocked (arrowhead in inset B). For further details, see Table 1.

We found the fraction of follicles showing dye-coupling between oocyte and follicle cells (Fig. 5) largely reduced in comparison to NIS by an antiserum directed against the intracellular C-terminus of Inx2 (AInx2Rb-CT(KLRH), *P *< 0.05) and also, but to a minor extent, by an antiserum directed against the intracellular loop of Inx2 (AInx2GP-CL, *P *< 0.05). Antisera directed against either Inx1, Inx3 or Inx4 did not reduce dye-coupling significantly when compared to NIS (*P *> 0.05; Table 1). Moreover, only Inx2-antisera were found to reduce dye-coupling in a concentration-dependent manner (not shown). These results demonstrate that Inx2 is involved in cell-cell communication by participating in gap-junction hemichannels residing in the oolemma. Although the difference observed between the CT-specific and the CL-specific antiserum was not significant (*P *> 0.05), the data suggest a prominent role of the C-terminus during closure of the pore.

### In-vitro development following inhibition of gap-junctional communication

**Figure 6:**
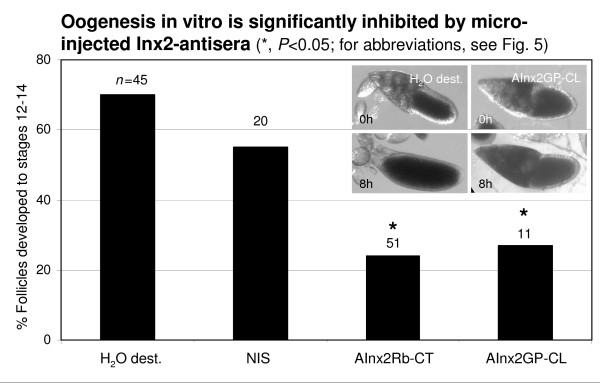
**Communication via Inx2-containing channels is essential for normal oogenesis in vitro**. Of the microinjected control follicles, 70% (H_2_O dest.) and 55% (NIS), respectively, developed in R-14 medium from stage 10b (0 h) to stages 12–14 (8 h, inset shows example). In contrast, in 76% (AInx2Rb-CT) and 73% (AInx2GP-CL), respectively, of the follicles microinjected with Inx2-antisera, oogenesis in vitro up to stage 12 was either incomplete or arrested in stage 10b (8 h, inset shows example). For further details, see Table 2.

We further analyzed the developmental capacities of follicles following microinjections of Inx2-antisera into the oocyte. In R-14 medium, stage-10b follicles are able to develop during 8 hours up to stage 14 [43,45]. When compared to NIS, in-vitro development was found to be inhibited in significant fractions of follicles microinjected with either AInx2Rb-CT(KLRH) or AInx2GP-CL (*P *< 0.05; Fig. 6). Since Inx4 is present in the oolemma only up to stage 8, AInx4Rb-CT served as a further control. The developmental capacities of follicles microinjected with AInx4Rb-CT were not significantly different from those obtained with NIS (*P *> 0.05; Table 2).

Most of the follicles microinjected with Inx2-antisera either stopped their development during stage 10b, which means that processes of follicle-cell differentiation, nurse-cell regression and oocyte growth were blocked (Fig. 6, inset; c.f. [41]). Or these follicles failed to complete choriogenesis normally, which becomes apparent by missing respiratory appendages, incomplete chorionic layers and sizeable nurse-cell remnants [43]. Therefore, it is obvious that communication between oocyte and follicle cells via Inx2-containing gap-junction channels is essential for normal follicle development through stages 10 to 14.

## Discussion

Although further proteins have been detected in *Drosophila *gap junctions [26,31,32], the main invertebrate gap-junction proteins are members of the innexin family [28,29]. During oogenesis and embryogenesis, the eight known *Drosophila *innexins show complex and overlapping expression patterns [37]. In the ovary, the mRNAs of innexins 1, 2, 3, 4 and 7 were found. Innexin mRNAs 1, 2, 3 and (to a minor extent) 7 were observed in the somatic follicle cells, especially in populations of migrating cells. In the oocyte and in the nurse cells, high amounts of innexin mRNAs 2 and 4 were detected, whereas the expression of innexin mRNAs 1, 3 and 7 was found to be rather low [37].

Since the mRNA-expression pattern only gives hints of the distribution and function of a protein, one aim of the present study was to reveal the cellular localization of Inx1, 2, 3 and 4 in the ovary. For Inx4, the early phases of localization and functioning have already been addressed: It is germ-line specific and plays an essential role during germ-cell differentiation in the germarium [13,38]. Concerning Inx1, 2 and 3, nothing was known so far. Moreover, it was not clear whether innexins form functional gap-junction channels in the ovary, and whether heteromeric channels (with hemichannels made of different innexins) and/or heterotypic channels (made of different hemichannels) may be involved.

During *Drosophila *embryogenesis, the roles of Inx1 and Inx2 have been analyzed in detail [12,14,39,46]. Both proteins participate in organizing embryonic epithelia by interacting with core proteins of adherens and septate junctions. By various experiments [40,47] it has been shown that Inx2 and Inx3, via their cytoplasmic C-terminal domains, form heterodimers, which are essential for the formation of heteromeric channels and for the integrity and polarity of the embryonic epidermis. Also in a *Xenopus*-oocyte expression system, Inx2 formed functional heteromeric channels with Inx3, whereas Inx2 alone, but not Inx3 alone, formed homotypic channels [48]. In this system, also Inx1 was found to form heteromeric channels with Inx2 and Inx3, respectively, whereas Inx4 only formed heterotypic channels with Inx2 [28].

Our immunoblot analysis using various innexin anti-peptide sera, that have been characterized before ([13,14,38-40] and R. Bauer, pers. com.), revealed the synthesis of Inx1, 2, 3 and 4 in the ovary. Although, in some cases, only weak bands at the calculated molecular masses were detected, further specific bands at lower molecular masses were revealed. Moreover, some antisera appeared to be better suited for immunohistochemistry than for immunoblotting or vice versa. Such phenomena are not unusual. For example, for Inx8 (Shak-B), which is not expressed in the ovary [37], only bands at lower molecular masses than deduced from the amino-acid sequence have been described [9].

Our immunhistochemical analysis revealed that Inx1 is predominantly localized to the baso-lateral domain of follicle cells, whereas Inx2 is positioned apico-laterally as well as apically between follicle cells and germ-line cells. Inx3 was observed laterally in follicle cells and also in nurse cells, and Inx4 was detected in the oolemma up to stage 8 and in nurse-cell membranes up to stage 12. While Inx2 and Inx3 are colocalized between soma cells, Inx2 and Inx4 are colocalized between soma and germ-line cells.

From the analysis of *Drosophila *mutants, Inx2 has been inferred to exert diverse structural functions [14,40,49]. Presumably, Inx2 is acting indirectly through the formation of membrane channels necessary for intercellular communication. We found that dye-coupling between oocyte and follicle cells was largely reduced by Inx2-antisera directed against both the intracellular C-terminus and the intracellular loop. Analyzing in vitro the developmental capacities of follicles following microinjections of Inx2-antisera revealed defects in follicle-cell differentiation, nurse-cell regression, oocyte growth and choriogenesis. Also in other invertebrates, communication via gap junctions has been found to exert influence on oogenesis [50].

Taken together, our results suggest that Inx1, 2, 3 and 4 are all involved in the formation of gap junctions during oogenesis. Based on immunostaining and microinjection experiments (as well as on heterologous expression [28,48]), we propose the existence of a variety of channels between the three cell types: (1) heteromeric channels made of Inx1/Inx3 between follicle cells, (2) homotypic channels made of Inx2 between follicle cells and oocyte, (3) homotypic channels made of Inx2 between germ-line cells, (4) heteromeric channels made of Inx2/Inx3 between follicle cells, (5) heteromeric channels made of Inx2/Inx3 between nurse cells, (6) heterotypic channels made of Inx2/Inx4 between follicle cells and germ-line cells (c.f. [13]), and (7) heterotypic channels made of Inx2/Inx4 between germ-line cells.

In previous studies, using light and electron microscopy, we have shown that antibodies against ductin bind to antigens located in the plasma membranes and in the cytoplasm of various *Drosophila *tissues, especially of ovarian follicles and embryos [31,32,51,52]. Ductin has been found in both vacuolar-type proton pumps and gap junctions [33]. By microinjection experiments, we have demonstrated that antibodies directed against presumed cytoplasmic regions of ductin block dye-coupling between germ-line and soma cells and exert influence on oogenesis as well as on embryogenesis [31,32].

## Conclusion

Thus, antibodies against both Inx2 and ductin inhibit gap-junctional communication between oocyte and follicle cells in *Drosophila *stage-10 follicles, and both proteins are present in the oolemma during this stage. In both cases, microinjected antibodies essentially affected normal follicle development. It remains to be clarified how innexins and ductins in gap junctions are related, and which molecules are to be exchanged between soma and germ-line cells.

## Abbreviations

AInx: anti-innexin; BSA: bovine serum albumine; CL: cytoplasmic loop; CT: C-terminus; DAPI: 4',6-diamidino-2-phenylindole; GP: guinea pig; Inx: innexin; LSM: laser-scanning microscope; LY: Lucifer Yellow CH; NIS: non-immune serum; PBS: phosphate buffered saline; Rb: rabbit; SDS-PAGE: sodium dodecyl sulphate polyacrylamide gel-electrophoresis; WFF: wide-field fluorescence.

## Authors' contributions

JB conceived the study, reviewed and analyzed the data and wrote the manuscript. JZ carried out the experiments under the supervision of JB and was involved in data analysis. Both authors read and approved the final manuscript.
